# Classification of breast cancer recurrence based on imputed data: a simulation study

**DOI:** 10.1186/s13040-022-00316-8

**Published:** 2022-12-07

**Authors:** Rahibu A. Abassi, Amina S. Msengwa

**Affiliations:** 1 Department of Natural Sciences, State University of Zanzibar, Zanzibar, Tanzania; 2 Department of Statistics, University of Dar es Salaam, Dar es Salaam, Tanzania

**Keywords:** Classification accuracy, Imputed data, Missing data mechanisms, Missingness percentages, Simulation

## Abstract

Several studies have been conducted to classify various real life events but few are in medical fields; particularly about breast recurrence under statistical techniques. To our knowledge, there is no reported comparison of statistical classification accuracy and classifiers’ discriminative ability on breast cancer recurrence in presence of imputed missing data. Therefore, this article aims to fill this analysis gap by comparing the performance of binary classifiers (logistic regression, linear and quadratic discriminant analysis) using several datasets resulted from imputation process using various simulation conditions. Our study aids the knowledge about how classifiers’ accuracy and discriminative ability in classifying a binary outcome variable are affected by the presence of imputed numerical missing data. We simulated incomplete datasets with 15, 30, 45 and 60% of missingness under Missing At Random (MAR) and Missing Completely At Random (MCAR) mechanisms. Mean imputation, hot deck, k-nearest neighbour, multiple imputations via chained equation, expected-maximisation, and predictive mean matching were used to impute incomplete datasets. For each classifier, correct classification accuracy and area under the Receiver Operating Characteristic (ROC) curves under MAR and MCAR mechanisms were compared. The linear discriminant classifier attained the highest classification accuracy (73.9%) based on mean-imputed data at 45% of missing data under MCAR mechanism. As a classifier, the logistic regression based on predictive mean matching imputed-data yields the greatest areas under ROC curves (0.6418) at 30% missingness while k-nearest neighbour tops the value (0.6428) at 60% of missing data under MCAR mechanism.

## Introduction

### Background

Missing data are frequently encountered in clinical research and they can negatively impact the research findings if not properly handled prior to data analysis due to their likelihood of biasing the results [1]. In clinical context, missing data may arise because of random errors caused by a measuring equipment, attrition due to social or natural processes as for instance death, non-response to some sensitive or unclear questions in a survey, and patients failing to report to a routine clinic [2]. Many researchers often discard incomplete cases during analysis stage, a technique known as complete case analysis (CCA). The CCA excludes any patients with at least one missing data value in statistical analysis, and thus reducing statistical power and introducing bias to the results [3]. The proper way to handle problems caused by missing data is to use imputation techniques [4] that estimate and replace missing values toyield complete dataset [5]. Imputation is an active area of research and several techniques including mean imputation, hot deck, k-nearest neighbour, various forms of multiple imputations by chained equations, predictive mean matching imputations, expected-maximization via bootstrapping imputations among others are used to handle with missing data under different conditions; however, there is no unique conclusive answer to which technique is best under a set of several conditions such as missing probabilities, missingness mechanisms, and patterns of missing data. An appropriate method to check the suitability and usefulness of imputation method is the use of simulation studies.

Prior to imputing missing data, it is important to understand the mechanism that generated the missing data. Every data value in a dataset has probability to be missing. Missing probabilities are governed by the process known as missing data mechanisms; they are divided into: Missing Completely At Random (MCAR), Missing At Random (MAR), and Missing Not At Random (MNAR) [6]. In MCAR, missing values neither depend on observed nor on unobserved values [3, 7] An example of MCAR data is when a test tube with patient’s urine sample is accidentally dropped and broken, yielding missing data associated with that laboratory testing. Data is said to be MAR when missing values depend only on observed values [3, 8, 9]. The reasons behind the missing data are associated with patient’s characteristics that are known [3]. An example of MAR data is when a patient deliberatively decide not to answer a certain question, especially if it is about his or her privacy [7]. The MNAR data occurs if the distribution of dataset containing missing values depends on missing values [9]. This implies that missing probability is associated to characteristics that the researcher cannot know about [8]. An example of MNAR data is when patients with low level of education tend to avoid questions concerning their educational status.

### Related work and analysis gap

Various studies have been conducted on distinct clinical datasets to classify breast recurrence under statistical and machine learning techniques. In some situations, simulation has been used along with classification process. Researchers use simulations to get answer to their specific questions about data analysis, and to assess models performance under specified desired conditions [10] Asimulation study on numerical data resulted from imputation techniques used to access the accuracy of prognostic models. Models’ discriminative ability to separate breast cancer patients with and without recurrence was determined by areas under ROC curves [11]. Another simulation study compared the performance of fully parametric imputations when imputation model was correctly specified and when miss specified using predictive mean matching and local residual draws imputation techniques [12].

The other study [13] evaluated the performance of different statistical and machine learning approaches in predicting recurrence of breast cancer from patients’ large real breast cancer dataset containing missing values. The study used discrimination and calibration measures to assess usefulness of the prognostic model. On the other hand, the comparison of imputation algorithms for building sensor data across several percentage of missing data was conducted by comparing the differences between real and imputed values through the use of Root Mean Squared Error and Mean Absolute Error estimates; its conclusion emphasized the necessity of identifying percentage of missingness prior to selecting proper imputation technique so as to reach plausible results [14]. Moreover, imputation techniques were used to evaluate the performance of model via discrimination, calibration, and effectiveness of classifiers in relation to time used to build a model in estimating the risk of unprovoked venous thrombo-embolism recurrence [15]. The focus was on diagnostic model development via multivariable logistic or prognostic model via survival regression analysis.

With due respect to mentioned related work, we aim to fill an analysis gap about performance of statistical classification techniques based on several datasets resulted from imputation techniques via simulation settings under various conditions. Our study observes the behaviour of three binary classifiers (logistic regression, linear and quadratic discriminant analysis) on numerical missing data based on simulation of real breast cancer dataset. Six imputed datasets across four different percentages of missing data and two missing data mechanisms were used for classification purpose. The goal was to assess how classifiers’ accuracy and discriminative ability (in classifying a binary outcome: breast cancer recurrence) are affected by the presence of imputed missing data values under various simulation conditions. We report the percentage accuracy and the areas under the ROC curves resulted from each classifier for 15, 30, 45 and 60% of missing data under MAR and MCAR mechanisms.

## Materials and methods

### Data descriprion

The study uses the simulated breast cancer datasets, each having 693 observations (with varying percentages of missing data contained in independent variables only). The variables used in this article were extracted from several previous breast cancer-related studies [10, 13, 16, 17]. The dependent or outcome variable namely ‘cancer recurrence’ has two response categories; ‘yes’ and ‘no’. The response ‘yes’ means a breast cancer comes back after recommended therapy, ‘no’ indicates that the cancer does not come back after respective therapy. The independent or predictor variables are: the age, heart rate, respiratory rate, body mass index, body surface area.

### Classification techniques

Classification techniques also known as classifiers are used to predict a categorical response for a case by assigning that case to a certain category. In this article, a binary logistic regression, linear, and quadratic discriminant classifiers [18, 19] were used to predict a group membership for breast cancer cases and classify to either response ‘recurrence’ or ‘non-recurrence’ based on patients’ demographic and clinical factors; ‘age, body mass index, body surface area, heart rate, and respiratory rate’.

#### Binary logistic regression

Binary logistic regression (BLR) model considers the probability that a response variable *Y* belongs to a particular group [18, 20]. In this article, the BLR model intended to predict and classify breast cancer cases to either recurrence or non-recurrence events given predictors. The model uses a *logit* or logistic function (a sigmoid function applied in binary classification). A sigmoid function takes predictors’ data (real numbers) and maps them to certain probability value (*ρ*_*i*_) between 0 and 1.

$$logit\left({\rho}_i\right)=\log \left(\frac{\rho_i}{1-{\rho}_i}\right)={\beta}_0+{\beta}_1{X}_{1i}+{\beta}_2{X}_{2i}+{\beta}_3{X}_{3i}+{\beta}_4{X}_{4i}+{\beta}_5{X}_{5i}$$

for *i* = 1, 2, 3, …, 693, *β*_0_ is a constant term of the model, *β*_*j*_ for *j* = 1, 2, …, 5 are the parameters of the model, *ρ*_*i*_ is the probability that *i*^*th*^ patient has cancer recurrence, *X*_*p*_ for *p* = 1, 2, …, 5 are the predictors in the model (age, heart rate, respiratory rate, body mass index, body surface area).

The patients’ probabilities (*ρ*_*i*_) of having breast cancer recurrence are estimated by the fitted logistic regression model. The (*ρ*_*i*_) are then used for classification of patients into either of the two categories of breast cancer response variable (recurrence or non-recurrence) according to the cut-off point of 0.5 for classification; a value greater than 0.5 is classified as ‘recurrence’ event, otherwise classified as ‘non-recurrence’ event for a patient *i* (*i*= 1,2, …, 693). Mathematically, the predicted probability for any patient *i* is given by *ρ*_*i*_(*x*):

$${\rho}_i(x)=\frac{\exp \left({\beta}_0+{\beta}_1{X}_{1i}+{\beta}_2{X}_{2i}+{\beta}_3{X}_{3i}+{\beta}_4{X}_{4i}+{\beta}_5{X}_{5i}\right)}{1+\exp \left({\beta}_0+{\beta}_1{X}_{1i}+{\beta}_2{X}_{2i}+{\beta}_3{X}_{3i}+{\beta}_4{X}_{4i}+{\beta}_5{X}_{5i}\right)}$$

#### Linear discriminant analysis

Linear discriminant analysis (LDA) is used to separate different sets cases and allocate new cases to previously defined groups [21]. It assumes that each group are drawn from Multivariate Normal distribution with group definite mean vector (μ) and covariance matrix (∑) [19]. As a classifier, LDA allocates cases **x** to group *k* provided that **x** is in falls inside the group or region *k* [22]. In this study we applied LDA to discriminate or separate the cases with breast cancer recurrence from those without recurrence by using the discrimination function, *δ*_*k*_(***x***). An assumption about equal covariance matrix, ∑ among the *k* = 2 groups was made to make discrimination function taking a linear form in ***x***. A Bayes’ theorem with grouping prior probabilities (π) is used to allocate **x** in group *k* for which a function.

$${\delta}_k\left(\boldsymbol{x}\right)={x}^T{\sum}^{-1}{\mu}_k-\frac{1}{2}{\mu}_k^T{\sum}^{-1}{\mu}_k+\log {\pi}_k\Big)$$ is largest [19].

#### Quadratic discriminant analysis (QDA)

As classifier, QDA is like LDA, it makes assumption about Multivariate Normality for observations within each group; however, it doesn’t assume common covariance matrix**,** i.e., each group assumed to have its own matrix. Bayes’ theorem is utilized to make prediction and allocate cases into respective groups [21, 23]. Nevertheless, . Based on this assumption, a case **x** assigned to a group *k* for which the function.

$${\delta}_k\left(\boldsymbol{x}\right)=-\frac{1}{2}{\left(x-{\mu}_k\right)}^T{\sum}_k^{-1}\left(x-{\mu}_k\right)-\frac{1}{2}\log \mid {\sum}_k\mid +\log {\pi}_k$$ is largest [19].

Since LDA and QDA the training and validation datasets, in this article we used 70 and 30% of the sample in each dataset as training and validation data respectively LDA is likely to perform better than QDA when the training cases are relatively few and hence reducing variance is vital. Meanwhile, QDA tends work better when number of training cases is very large, as a result the classifier’s variance does not harm, also it is a recommended classifier if covariance matrix is clearly common among the *k* groups [19]. .

### Simulation settings

The simulation experiment was conducted to compare the performance of imputation methods and classifiers in presence of imputed missing values across different simulation conditions. The simulation of data was based on real breast cancer dataset of size 693 containing both observed and missing values for variables; *X*_1_, *X*_2_, …, *X*_6_ respectively, denoting the age, heart rate, respiratory rate, body mass index, body surface area, and recurrence of breast cancer. Data were sampled with fixed mean vector and covariance matrix of all variables while varying the percentages of missing data (*i* = 1 : 4), changing missingness mechanisms (*j* = 1 : 2), and imputation methods (*k* = 1 : 6) producing a total of 4 × 2 × 6 = 48 simulation conditions. The experimental design was built in four steps: (1) Generating complete data set, (2) Amputation (making incomplete data sets from the completed one), (3) Imputation (to fill-in missing data values), and (4) Evaluate the performance of each imputation techniques.

In step (1) we generated a complete dataset with size *N* = 693 observations from multivariate normal distribution [24, 25] with vector of means (*μ*), and a positive definite covariance matrix (∑) given below; archived by the application of ‘*mvrnorm*’ function in the package ‘*MASS*’ [26] of R statistical program.

$$\mu =\left[\begin{array}{c}\begin{array}{c}51.00\\ {}97.89\\ {}20.84\end{array}\\ {}27.71\\ {}\kern0.5em 1.69\\ {}\kern0.75em 0.31\end{array}\right]$$

$$\Sigma =\left[\begin{array}{cccccc}169.3& \kern1em 1.8& \kern0.5em 3.0& \kern0.5em 2.0& -0.2&\ 0.1\\ {}\kern1em 1.8& 486.7& \kern0.5em 9.7& -4.7& \kern0.5em -0.2& \kern0.5em 1.1\\ {}\kern1em 3.0& \kern1em 9.7&\ 37.5& -0.5& -0.01& \kern0.5em 0.2\\ {}\kern1em 2.0& -4.7& -0.5&\ 43.6& \kern1.5em 0.9& -0.2\\ {}-0.2& -0.2& -0.01& \kern1em 0.9& \kern1.5em 0.1&\ 0.01\\ {}\kern1em 0.1& \kern1em 1.1& \kern1.25em 0.2& -0.2& \kern1em 0.01& \kern0.5em 0.2\end{array}\right]$$

Step (2) performed the ‘amputation’ (i.e., generating missing data values from the complete data set, in step 1). This involved creating incomplete data sets with varying percentages of missing data (15, 30, 45, and 60%) and two missing data mechanisms (MAR and MCAR). The R function, ‘*amput*’ [27] was applied to meet this purpose. The third step was to impute the created missing data sets using the following distinct imputation techniques:

#### Mean imputation

The idea based on this approach is to replace missing data values by the variable’s average score from observed data [3, 13, 28]. Theoretically, the mean imputation is more appropriate when the amount of missing data is small whilst the sample size is large [7]. Mean imputation also known as ‘series mean’ (SMEAN) is calculated as $\sum_{\boldsymbol{i}=\textbf{1}}^{\boldsymbol{n}}{\boldsymbol{x}}_{\boldsymbol{i}}/\boldsymbol{n}$ where ***x***_***i***_ is a numerical variable and ***i*** = **1**, **2**, …, ***n***; number of patients with observed covariate’s data values.

#### Hot deck imputation

This technique replaces each missing data in a variable by the observed data from a patient with ‘identical response’or data in the same variable [28]. The patient with missing data (un-respondent) is known as ‘recipient’ while the responded one with observed data is called the ‘donor’ [7, 29]. This method works better with MCAR or MAR data mechanisms [7]. Consider the values x_i_ = (x_i1_, …, x_ip_ ) for subject i of p covariates. For a matching recipient i and a donor j, the proximity of potential candidate donors to recipients is defined by maximum deviation given by *D*_(*i*, *j*)_ = max_*k*_|*x*_*ik*_ − *x*_*jk*_|, for nicely scaled *x*_*k*_ so that the comparability of difference) can be made [29]. In this study the hot deck imputation was implemented by using function ‘hot deck’ from the ‘VIM’ (Visualization and Imputation of Missing Values) package [30] in R statistical software (version 3.6.3).

#### K-nearest neighbour (KNN)

A non-parametric approach used to impute missing data by averaging its neighbouring observed data [14]. The approach is donor-based in which imputed values are either measured as a single records in the dataset (1-NN) or as an average value obtained from k records (k-NN) [31]. The distance between two observations that is used to define the nearest neighbours is defined as${D}_{ij}=\frac{\sum_{k=1}^P{w}_k{\tau}_{i,j,k}}{\sum_{k=1}^P{w}_k}$, where *w*_*k*_ is the weight and *τ*_*i*, *j*, *k*_ is the contribution of *k*^*th*^ variable. The ratio of absolute distance to range is used for *τ*_*i*, *j*, *k*_ of continuous variables; ${\tau}_{i,j,k}=\frac{\mid {x}_{i,k}-{x}_{j,k}\mid }{r_k}$, whereas *x*_*i*, *k*_ is a value of *k*^*th*^ variable of *i*^*th*^ observation and *r*_*k*_ is the range of *k*^*th*^ variable (30). In this study, the hot deck imputation method was performed by using function ‘hot deck’ from the ‘VIM’ (Visualization and Imputation of Missing Values) package [30] in R statistical software (version 3.6.3).

#### Multiple imputation by chained equations (MICE)

MICE Replaces each missing data with set of *P* acceptable values [32]. The method works with MCAR or MAR missing data mechanisms. The technique helps to remove potential selection bias which would result if observations with missing values were deleted from the dataset. Moreover, the chance of getting biased standard errors is also reduced [7, 8]. The procedure for the multiple imputations involves the following three steps: ‘imputing data *m* times, analysis of *m* imputed datasets, and pooling of results’ [6]. We applied R statistical software to perform these three steps in MICE by storing the results from each in the three special classes; *mids* (multiple imputed data sets), *mira* (multiple imputed repeated analysis), and *mipo* (multiple imputed pooled results) [33].

#### Predictive mean matching (PMM)

The PMM utilizes both parametric and non-parametric approaches to impute missing data. The parametric aspect, establishes a predictive mean value corresponding to each observation in data. These predictive means are then used to match complete and incomplete observations during imputation process. The non-parametric stage applies the method of Nearest Neighbour Donor to produce original data value from non-missing observation having nearest predictive mean distance close to missing one so as to impute a missing data value [34, 35]. The function and package ‘mice’ in R statistical software [33] was used to perform the PMM imputation five times, storing results from five complete datasets, and combining the results from five analysed datasets.

#### Expected maximization via bootstrapping (EMB)

The EMB is a bootstrap-based algorithm used to impute missing data multiple times. It utilizes existing sample data of size *n* to make new *M* samples of size *n* with replacement [36]. Since EMB is a multiple imputations-based procedure, it performs better under MAR assumption. It is based on E-M algorithm, summarized as follows: Firstly, the EM (Expectation-Maximisation) algorithm make use of certain distribution and propose starting/initial values for mean, μ and covariance matrix, ∑ that are then used to calculate an expected value of model’s likelihood. This likelihood is maximised and parameters of the model are estimated and updated. The steps of expectation and maximisation are repeated until convergence of the values is reached [36, 37]. The implementation of EMB in done in Amelia II program starts with bootstrapping an incomplete dataset to generate several bootstrapped datasets, followed by E-M process of these data to imputed datasets and then the imputed datasets are analysed separately by standard statistical method, and the results are combine to provide single final results.

### Evaluation of imputation methods

The evaluation of applied imputation techniques was through comparison of Root Mean Squared Errors (RMSE) obtained from each imputation techniques under various simulations conditions. For each imputation method, we also report classification accuracy resulted from three classification methods via estimated area under the Receiver Operating Characteristics (ROC) curves resulted from logistic regression using imputed data sets at 15, 30, 45, and 60% missing data under MAR and MCAR mechanisms.

## Results

The average values of RMSE for each imputation method (Table 1) tend to increase as percentages of missing data are increased. This reflects the reduction of efficiency of imputations as missing percentages increase in datasets. Under MAR, the EMB technique attained the least RMSE values at lowest percentage (15%) of missing data followed by PMM compared to other methods. The SMEAN acquired lowest values of RMSE followed by PMM at highest percentage of missing data (60%), this indicates that the PMM is more efficient low and high percentages of missing data for MAR and MCAR mechanisms.

**Table 1:** RMSE for imputation methods across various percentages of missing data

Missingness %	SMEAN	HD	KNN	PMM	MICE	EMB
RMSE for imputation methods under MAR						
15	6.40	6.10	5.90	5.80	6.00	5.60
30	10.40	10.90	10.50	10.80	10.90	11.10
45	11.20	11.20	11.30	11.30	11.60	10.80
60	12.80	13.10	12.70	12.70	12.80	13.30
RMSE for imputation methods under MCAR						
15	6.90	7.10	7.00	7.00	8.00	7.10
30	9.30	8.70	8.60	9.80	9.70	10.10
45	11.00	11.30	11.20	11.20	11.10	11.20
60	12.70	13.20	13.00	12.50	12.80	13.10

**Fig. 1:**
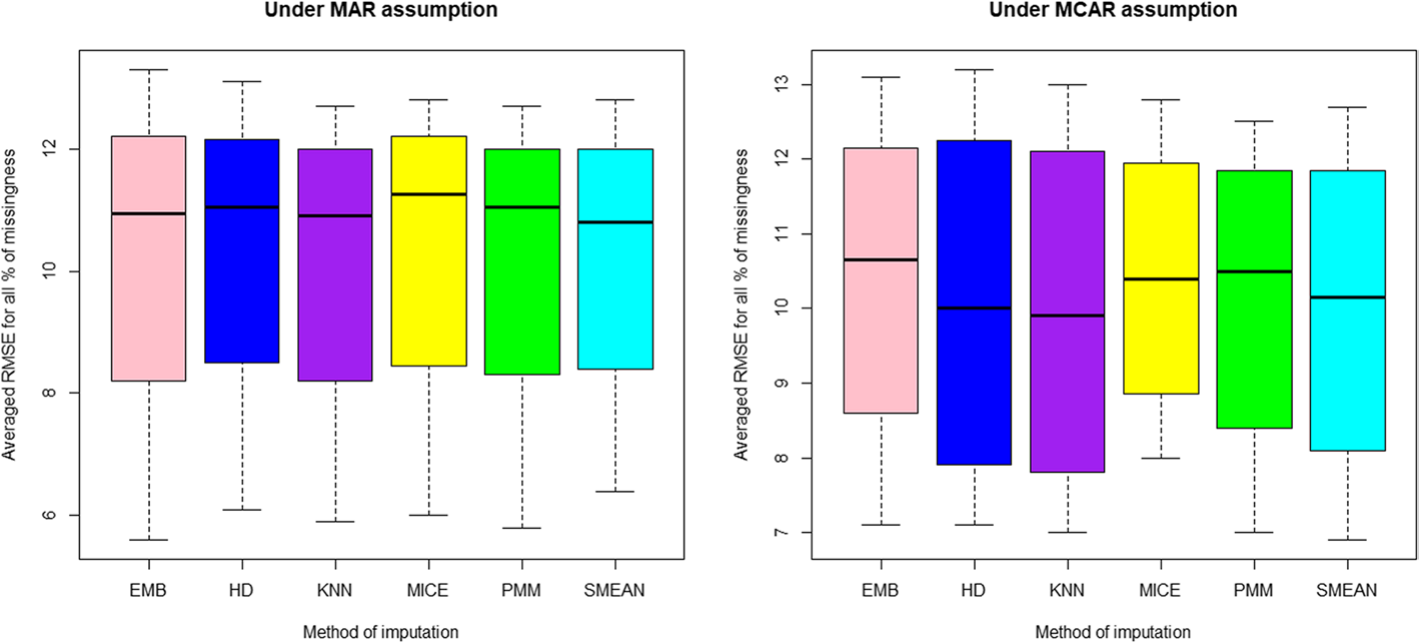
Distribution of averaged RMSE under MAR and MCAR conditions

The distributions of average RMSE (Fig. 1) for imputation methods under MCAR are much close to symmetric shape as compared those under MAR mechanism, meaning that the MCAR mechanism yields imputation results whose central tendency measures are close to each other, implying a little difference among them under the current study and scenario. All areas under the ROC curves (Table 2) from binary logistic regression classifier using each imputed dataset under four percentages of missing data and two missing data mechanisms are above 0.5 implies that all methods can discriminate recurrence from non-recurrence cases.

**Table 2:** Areas under ROC curves from logistic regression based on each imputation methods with MAR and MCAR assumptions and 15, 30, 45, 60% of missing data

Method	Areas under ROC for MAR				Areas under ROC for MCAR			
	15%	30%	45%	60%	15%	30%	45%	60%
SMEAN	0.6015	0.6064	0.6064	0.5962	0.6090	0.6044	0.6153	0.6011
HD	0.5914	0.6096	0.5730	0.6334	0.6110	0.6097	0.5955	0.5913
KNN	0.6081	0.6041	0.5921	0.6041	0.6083	0.6071	0.6328	0.6428
PMM	0.6134	0.6418	0.6343	0.6164	0.6157	0.6062	0.6374	0.6093
MICE	0.6065	0.6275	0.6245	0.6060	0.6057	0.6165	0.6249	0.6229
EMB	0.6014	0.6357	0.5993	0.5733	0.6000	0.6027	0.6318	0.6099

**Fig. 2:**
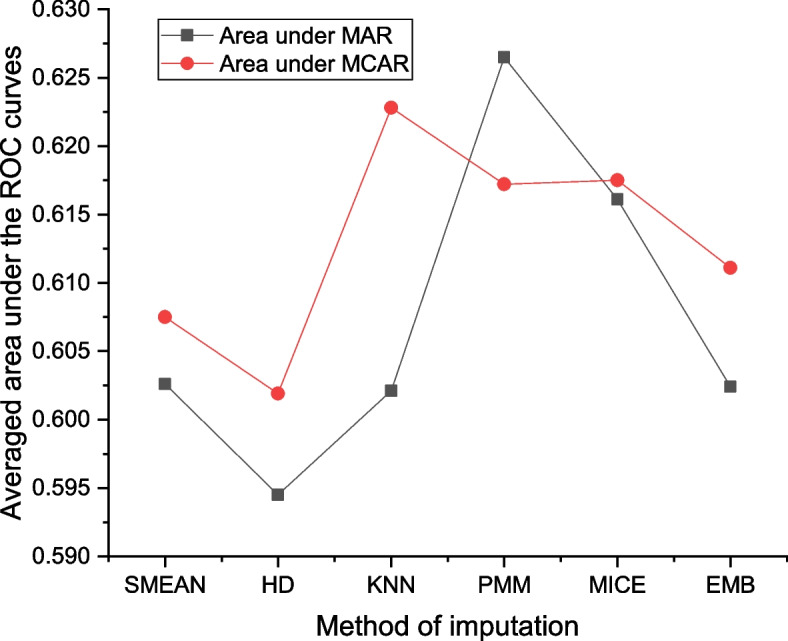
Averaged area under the ROC curves

Across all percentages and missing mechanisms, the maximum area under ROC curve is 0.6428 from KNN at 60% missing data with MCAR, followed by PMM at 30% under MAR. Under MAR, the PMM attains the highest values of areas under at 15, 30, and 45% of missing data while the HD appeared top at 60% of missing data; again, the PMM yields the greatest areas under ROC curves at 15 and 45%, while KNN top the value at 60% and MICE at 30% under MCAR assumption. This information and Fig. 2 suggests that the PMM and KNN are more plausible based on discriminating the recurrence and non-recurrence cases.

We observe (Table 3) that by, using a MAR mechanism for the 15% missing data, the series mean imputation (SMEAN) yields better classification accuracy via both classifiers; binary logistic regression (BLR) got the top result (70.42%) amongst the three classifiers. For the MCAR mechanism at 15%, the expected-maximisation via bootstrapping (EMB) imputation method yields best accuracy of 71.60% using linear discriminant analysis (LDA) classifier. At 30% of missing data and MCAR mechanism, the linear discriminant analysis (LDA) via SMEAN achieved best classification accuracy (72.9%) while the BLR produced highest accuracy (71.4%) via SMEAN at 30% with MAR mechanism. Using 45% of missing data, the LDA classifier indicates best classification accuracy (73.9%) under MCAR mechanism, meanwhile, the 60% missing rate reveals that BLR via SMEAN produces best accuracy (72.3%) under MAR mechanism.

**Table 3:** Classification accuracy (%) for each imputation method using LDA, QDA, and BLR classifiers under both MAR and MCAR mechanisms

Missing data %	Imputation Method	Accuracy under (%) MAR			Accuracy under (%) MCAR		
		LDA	QDA	BLR	LDA	QDA	BLR
15%	SMEAN	70.14	69.12	70.42	71.10	68.20	71.00
	HD	68.72	67.77	69.70	69.70	68.20	70.10
	KNN	68.25	68.25	69.55	67.50	67.50	70.00
	PMM	68.70	68.25	69.30	71.10	68.70	70.00
	MICE	68.28	67.30	69.12	68.20	67.70	70.10
	EMB	65.33	65.33	69.84	71.60	69.70	69.60
30%	SMEAN	70.10	68.20	71.40	72.90	72.00	70.80
	HD	68.20	67.20	68.80	69.80	67.40	69.70
	KNN	67.80	67.80	70.80	70.60	69.70	70.30
	PMM	70.80	67.90	70.40	71.10	68.70	69.30
	MICE	70.70	70.70	64.80	71.60	72.50	69.70
	EMB	68.20	66.40	71.10	69.70	68.20	69.40
45%	SMEAN	73.50	73.90	73.40	73.90	72.00	72.40
	HD	69.19	68.25	70.56	69.10	68.10	69.80
	KNN	72.00	72.90	72.20	69.70	69.70	70.70
	PMM	66.80	69.20	70.30	70.60	70.10	70.60
	MICE	68.20	67.80	69.30	69.20	65.90	70.30
	EMB	67.30	65.90	69.30	68.70	69.20	68.70
60%	SMEAN	70.10	70.10	72.30	72.00	69.70	71.70
	HD	68.20	67.20	68.80	67.30	67.80	69.10
	KNN	68.20	68.70	69.60	65.90	63.50	68.90
	PMM	68.20	66.40	69.80	64.90	66.40	68.50
	MICE	68.70	65.40	69.40	67.80	68.70	69.10
	EMB	67.80	68.20	68.80	66.50	67.50	68.30

## Discussion

The article intended to assess classifiers’ accuracy and discriminative ability in classifying breast cancer recurrence using simulation approach based on imputation methods. Before assessing the accuracy and discrimination of classifiers, we used the average RMSE to assess the performance of imputation methods used to fill-in missing data generated through simulation of breast cancer datasets under various percentages (15, 30, 45 and 60%) of missing data and missing mechanisms (MAR and MCAR). It was observed that the performance of imputation approaches were decreased with increased missing percentages since the values of RMSE are direct proportion to percentages of missing data under both missing data mechanisms; MAR and MCAR. In addition, the KNN and PMM methods performed better at low and high percentages of missing data for both MAR and MCAR mechanisms. This finding is in line with Javadh et al. [38] and Kleinke at al [39].

It was observed that the performance of imputation approaches were decreased with increased missing percentages since the values of RMSE are direct proportion to percentages of missing data under both missing data mechanisms; MAR and MCAR. In addition, the PMM method performed better at low and high percentages of missing data for both MAR and MCAR mechanisms.

The assessment of performance of classification of three classifiers; binary logistic regression (BLR), linear discriminant analysis (LDA), and quadratic discriminant analysis (QDA) across 15, 30, 45 and 60% of missing data and missing mechanisms (MAR and MCAR) from each applied imputation method (Table 3). The highest classification accuracy (73.9%) was achieved via LDA based on mean-imputed data (at 45% missingness with MCAR mechanism), and the minimum was 64.8% from BLR (at 30% missingness with MAR mechanism). This finding implies that the best classifier is LDA under MCAR data. This observation agree the information revealed in Fig. 1 where it was worth noting that the MCAR mechanism provided much alike imputed values than MAR and hence making sense when the mechanism lead to slight better classification accuracy via LDA. However, Ghorbani and Desmarais [40] claim that the performance of imputation techniques considerably changes between various classifiers under different percentages of missing data while EMB gave best classification accuracy across all percentages of missing data.

The binary logistic regression was applied to compare the discriminative ability (how observations from ‘recurrence group’ are separated from ‘non-recurrence’ group) through the magnitude of area under the ROC across 15, 30, 45 and 60% of missing data and missing mechanisms, MAR and MCAR from each applied imputation method (Table 2). The model revealed that k-nearest neighbour (KNN) and predictive mean matching (PMM) imputations provide close maximum area under ROC curves, 0.6428 (at 60% missingness with MAR mechanism) and 0.6418 (at 30% of missing data with MCAR mechanism) respectively. A study [41] demonstrated the ability to predict or discriminate the recurrence from non-recurrence cases of breast cancer prior to ‘neo-adjuvant chemotherapy’ treatment yield area under ROC curve of 75%; a difference of about 11% compared to ours. This implies that the imputed data via KNN and PMM are most plausible to enable classifiers to separate breast cancer recurrence cases for at least 64%.

## Conclusions

Based on simulation study under various percentages and mechanisms of missing numerical data, the article’s conclusions are focused on three parts. First, a predictive mean matching is most plausible imputation methods for numerical variables; second, the linear discriminant analysis provides better classification accuracy over binary logistic regression and quadratic discriminant analysis for dichotomous response variable; third, logistic regression model is able to separate or discriminate breast cancer recurrence from non-recurrence cases for at least 64%. The future breast cancer classification-related studies may focus on the of imputing longitudinal survival truncated data prior to classification of breast cancer recurrence.

## Data Availability

The datasets used and/or analysed during the current study are available from the corresponding author on reasonable request.

## Abbreviations

BSA: Body Surface Area
BMI: Body Mass Index
EMB: Expectation-Maximization via Bootstrap
MAR: Missing At Random
MICE: Multiple Imputations by Chained Equations
MCAR: Missing Completely At Random
NMAR: Not Missing At Random
ROC: Receiver Operating Characteristics
KNN: K-Nearest Neighbour
VIM: Visualization and Imputation of Missing Values
PMM: Predictive Mean Matching
R: R statistical software
